# Supplementary material for: The Prediction of Cu(II) Adsorption Capacity of Modified Pomelo Peels Using the PSO-ANN Model
Source: Molecules. 2023 Oct 6;28(19):6957. doi: 10.3390/molecules28196957 (PMC10574590; doi:10.3390/molecules28196957)
Supplement: Supplementary file 1 [file molecules-28-06957-s001.zip › molecules-2636982-supplementary.pdf]

# Supplementary Data

# Prediction of Cu(II) Adsorption Capacity by Modified Pomelo Peels Using the PSO-ANN Model

**Mengqing Jiao [1], Johan Jacquemin [2], Ruixue Zhang [1], Nan Zhao [1,3,*] and Honglai Liu [3]**

[1] Hebei Key Laboratory of Green Development of Rock and Mineral Materials, Hebei GEO University, Shijiazhuang 050031, China; jmq15931164981@163.com (M.J.); zhangruixue77@163.com (R.Z.)
[2] Materials Science and Nano-Engineering MSN Department, Mohammed VI Polytechnic University, Lot 660-Hay Moulay Rachid, Ben Guerir 43150, Morocco; johan.jacquemin@um6p.ma
[3] School of Chemistry and Molecular Engineering, East China University of Science and Technology, Shanghai 200237, China; hlliu@ecust.edu.cn
* Correspondence: zhaonan_63@163.com

**Content:**

The training data used for the ANN model are tabulated in Table S1. The testing data used for the ANN model are tabulated in Table S2.

**Table S1** Training Data used for Developing the ANN Model during this Work

| No. | pH | *T* (K) | $C_o$ (mg/L) | t (min) | $C_e$ (mg/L) | $q_e$ (mg/L) | *R* (%) |
|---|---|---|---|---|---|---|---|
| 1 | 2.0 | 298.15 | 20 | 60 | 2.7930 | 4.3018 | 86.04% |
| 2 | 2.2 | 298.15 | 20 | 60 | 2.3079 | 4.4230 | 88.46% |
| 3 | 2.6 | 298.15 | 20 | 60 | 1.6641 | 4.5840 | 91.68% |
| 4 | 2.8 | 298.15 | 20 | 60 | 1.4752 | 4.6312 | 92.62% |
| 5 | 3.0 | 298.15 | 20 | 60 | 1.3548 | 4.6613 | 93.23% |
| 6 | 3.2 | 298.15 | 20 | 60 | 1.2877 | 4.6781 | 93.56% |
| 7 | 3.6 | 298.15 | 20 | 60 | 1.2530 | 4.6868 | 93.74% |
| 8 | 3.8 | 298.15 | 20 | 60 | 1.2552 | 4.6862 | 93.72% |
| 9 | 4.0 | 298.15 | 20 | 60 | 1.2502 | 4.6875 | 93.75% |
| 10 | 4.2 | 298.15 | 20 | 60 | 1.2271 | 4.6932 | 93.86% |
| 11 | 4.6 | 298.15 | 20 | 60 | 1.1543 | 4.7114 | 94.23% |
| 12 | 4.8 | 298.15 | 20 | 60 | 1.1246 | 4.7188 | 94.38% |
| 13 | 5.0 | 298.15 | 20 | 60 | 1.1129 | 4.7218 | 94.44% |
| 14 | 5.2 | 298.15 | 20 | 60 | 1.1270 | 4.7183 | 94.37% |
| 15 | 5.6 | 298.15 | 20 | 60 | 1.2284 | 4.6929 | 93.86% |
| 16 | 5.8 | 298.15 | 20 | 60 | 1.3106 | 4.6723 | 93.45% |
| 17 | 6.0 | 298.15 | 20 | 60 | 1.4104 | 4.6474 | 92.95% |
| 18 | 6.2 | 298.15 | 20 | 60 | 1.5250 | 4.6188 | 92.38% |
| 19 | 6.6 | 298.15 | 20 | 60 | 1.7883 | 4.5529 | 91.06% |
| 20 | 6.8 | 298.15 | 20 | 60 | 1.9319 | 4.5170 | 90.34% |
| 21 | 7.0 | 298.15 | 20 | 60 | 2.0799 | 4.4800 | 89.60% |
| 22 | 5.0 | 298.15 | 20 | 10 | 3.4139 | 4.1465 | 82.93% |
| 23 | 5.0 | 298.15 | 20 | 12 | 2.8963 | 4.2759 | 85.52% |
| 24 | 5.0 | 298.15 | 20 | 16 | 2.1488 | 4.4628 | 89.26% |
| 25 | 5.0 | 298.15 | 20 | 18 | 1.8963 | 4.5259 | 90.52% |
| 26 | 5.0 | 298.15 | 20 | 20 | 1.7096 | 4.5726 | 91.45% |
| 27 | 5.0 | 298.15 | 20 | 22 | 1.5773 | 4.6057 | 92.11% |
| 28 | 5.0 | 298.15 | 20 | 26 | 1.4312 | 4.6422 | 92.84% |
| 29 | 5.0 | 298.15 | 20 | 28 | 1.3948 | 4.6513 | 93.03% |
| 30 | 5.0 | 298.15 | 20 | 30 | 1.3678 | 4.6581 | 93.16% |

| No. | pH | $T$ (K) | $C_o$ (mg/L) | t (min) | $C_e$ (mg/L) | $q_e$ (mg/L) | $R$ (%) |
|---|---|---|---|---|---|---|---|
| 31 | 5.0 | 298.15 | 20 | 32 | 1.3408 | 4.6648 | 93.30% |
| 32 | 5.0 | 298.15 | 20 | 36 | 1.2863 | 4.6784 | 93.57% |
| 33 | 5.0 | 298.15 | 20 | 38 | 1.2611 | 4.6847 | 93.69% |
| 34 | 5.0 | 298.15 | 20 | 40 | 1.2390 | 4.6903 | 93.81% |
| 35 | 5.0 | 298.15 | 20 | 42 | 1.2209 | 4.6948 | 93.90% |
| 36 | 5.0 | 298.15 | 20 | 46 | 1.1942 | 4.7015 | 94.03% |
| 37 | 5.0 | 298.15 | 20 | 48 | 1.1840 | 4.7040 | 94.08% |
| 38 | 5.0 | 298.15 | 20 | 50 | 1.1747 | 4.7063 | 94.13% |
| 39 | 5.0 | 298.15 | 20 | 52 | 1.1655 | 4.7086 | 94.17% |
| 40 | 5.0 | 298.15 | 20 | 56 | 1.1440 | 4.7140 | 94.28% |
| 41 | 5.0 | 298.15 | 20 | 58 | 1.1301 | 4.7175 | 94.35% |
| 42 | 5.0 | 298.15 | 4 | 60 | 0.9098 | 0.7726 | 77.26% |
| 43 | 5.0 | 298.15 | 5 | 60 | 0.9432 | 1.0142 | 81.14% |
| 44 | 5.0 | 298.15 | 7 | 60 | 0.9819 | 1.5045 | 85.97% |
| 45 | 5.0 | 298.15 | 8 | 60 | 0.9918 | 1.7521 | 87.60% |
| 46 | 5.0 | 298.15 | 9 | 60 | 0.9984 | 2.0004 | 88.91% |
| 47 | 5.0 | 298.15 | 10 | 60 | 1.0042 | 2.2490 | 89.96% |
| 48 | 5.0 | 298.15 | 12 | 60 | 1.0228 | 2.7443 | 91.48% |
| 49 | 5.0 | 298.15 | 13 | 60 | 1.0394 | 2.9902 | 92.00% |
| 50 | 5.0 | 298.15 | 14 | 60 | 1.0588 | 3.2353 | 92.44% |
| 51 | 5.0 | 298.15 | 15 | 60 | 1.0778 | 3.4806 | 92.81% |
| 52 | 5.0 | 298.15 | 17 | 60 | 1.1018 | 3.9746 | 93.52% |
| 53 | 5.0 | 298.15 | 18 | 60 | 1.1063 | 4.2234 | 93.85% |
| 54 | 5.0 | 298.15 | 19 | 60 | 1.1090 | 4.4727 | 94.16% |
| 55 | 5.0 | 298.15 | 22 | 60 | 1.1296 | 5.2176 | 94.87% |
| 56 | 5.0 | 298.15 | 23 | 60 | 1.1414 | 5.4646 | 95.04% |
| 57 | 5.0 | 298.15 | 24 | 60 | 1.1546 | 5.7114 | 95.19% |
| 58 | 5.0 | 298.15 | 25 | 60 | 1.1680 | 5.9580 | 95.33% |
| 59 | 5.0 | 298.15 | 27 | 60 | 1.1928 | 6.4518 | 95.58% |
| 60 | 5.0 | 298.15 | 28 | 60 | 1.2025 | 6.6994 | 95.71% |
| 61 | 5.0 | 288.15 | 20 | 60 | 1.4336 | 4.6416 | 92.83% |
| 62 | 5.0 | 289.15 | 20 | 60 | 1.3503 | 4.6624 | 93.25% |
| 63 | 5.0 | 291.15 | 20 | 60 | 1.2347 | 4.6913 | 93.83% |
| 64 | 5.0 | 292.15 | 20 | 60 | 1.1976 | 4.7006 | 94.01% |

| No. | pH | *T* (K) | $C_o$ (mg/L) | t (min) | $C_e$ (mg/L) | $q_e$ (mg/L) | *R* (%) |
|---|---|---|---|---|---|---|---|
| 65 | 5.0 | 293.15 | 20 | 60 | 1.1712 | 4.7072 | 94.14% |
| 66 | 5.0 | 294.15 | 20 | 60 | 1.1528 | 4.7118 | 94.24% |
| 67 | 5.0 | 296.15 | 20 | 60 | 1.1310 | 4.7172 | 94.34% |
| 68 | 5.0 | 297.15 | 20 | 60 | 1.1227 | 4.7193 | 94.39% |
| 69 | 5.0 | 299.15 | 20 | 60 | 1.0997 | 4.7251 | 94.50% |
| 70 | 5.0 | 301.15 | 20 | 60 | 1.0661 | 4.7335 | 94.67% |
| 71 | 5.0 | 302.15 | 20 | 60 | 1.0478 | 4.7381 | 94.76% |
| 72 | 5.0 | 303.15 | 20 | 60 | 1.0301 | 4.7425 | 94.85% |
| 73 | 5.0 | 304.15 | 20 | 60 | 1.0138 | 4.7466 | 94.93% |
| 74 | 5.0 | 306.15 | 20 | 60 | 0.9858 | 4.7536 | 95.07% |
| 75 | 5.0 | 307.15 | 20 | 60 | 0.9739 | 4.7565 | 95.13% |
| 76 | 5.0 | 308.15 | 20 | 60 | 0.9633 | 4.7592 | 95.18% |
| 77 | 5.0 | 309.15 | 20 | 60 | 0.9539 | 4.7615 | 95.23% |
| 78 | 5.0 | 311.15 | 20 | 60 | 0.9384 | 4.7654 | 95.31% |
| 79 | 5.0 | 312.15 | 20 | 60 | 0.9321 | 4.7670 | 95.34% |
| 80 | 5.0 | 313.15 | 20 | 60 | 0.9267 | 4.7683 | 95.37% |

**Table S2.** Testing Data used for Validation of the ANN Model during this Work

| No. | pH | *T* (K) | $C_o$ (mg/L) | t (min) | $C_e$ (mg/L) | $q_e$ (mg/L) | *R* (%) |
|---|---|---|---|---|---|---|---|
| 1 | 2.4 | 298.15 | 20 | 60 | 1.9366 | 4.5158 | 90.32% |
| 2 | 3.4 | 298.15 | 20 | 60 | 1.2588 | 4.6853 | 93.71% |
| 3 | 4.4 | 298.15 | 20 | 60 | 1.1918 | 4.7021 | 94.04% |
| 4 | 5.4 | 298.15 | 20 | 60 | 1.1663 | 4.7084 | 94.17% |
| 5 | 6.4 | 298.15 | 20 | 60 | 1.6518 | 4.5871 | 91.74% |
| 6 | 5.0 | 298.15 | 20 | 14 | 2.4784 | 4.3804 | 87.61% |
| 7 | 5.0 | 298.15 | 20 | 24 | 1.4883 | 4.6279 | 92.56% |
| 8 | 5.0 | 298.15 | 20 | 34 | 1.3133 | 4.6717 | 93.43% |
| 9 | 5.0 | 298.15 | 20 | 44 | 1.2062 | 4.6985 | 93.97% |
| 10 | 5.0 | 298.15 | 20 | 54 | 1.1556 | 4.7111 | 94.22% |
| 11 | 5.0 | 298.15 | 6 | 60 | 0.9665 | 1.2584 | 83.89% |
| 12 | 5.0 | 298.15 | 11 | 60 | 1.0116 | 2.4971 | 90.80% |
| 13 | 5.0 | 298.15 | 16 | 60 | 1.0929 | 3.7268 | 93.17% |
| 14 | 5.0 | 298.15 | 21 | 60 | 1.1197 | 4.9701 | 94.67% |
| 15 | 5.0 | 298.15 | 26 | 60 | 1.1811 | 6.2047 | 95.46% |
| 16 | 5.0 | 290.15 | 20 | 60 | 1.2848 | 4.6788 | 93.58% |
| 17 | 5.0 | 295.15 | 20 | 60 | 1.1403 | 4.7149 | 94.30% |
| 18 | 5.0 | 300.15 | 20 | 60 | 1.0837 | 4.7291 | 94.58% |
| 19 | 5.0 | 305.15 | 20 | 60 | 0.9991 | 4.7502 | 95.00% |
| 20 | 5.0 | 310.15 | 20 | 60 | 0.9457 | 4.7636 | 95.27% |
